# Doctor-patient communication during the Corona crisis – web-based interactions and structured feedback from standardized patients at the University of Basel and the LMU Munich

**DOI:** 10.3205/zma001477

**Published:** 2021-04-15

**Authors:** Wolf Langewitz, Ulrich Pleines Dantas Seixas, Sabina Hunziker, Christoph Becker, Martin R. Fischer, Alexander Benz, Bärbel Otto

**Affiliations:** 1 University Hospital Basel, Medical Communication and Psychosomatic Medicine, Basel, Switzerland; 2 University of Basel, Faculty of Medicine, Basel, Switzerland; 3 LMU Munich, University Hospital, Institute for Medical Education, Munich, Germany; 4 Ludwig Maximilian University Munich (LMU), Institute of Medical Psychology, Munich, Germany; 5 LMU Munich, Institute for Medical Education, University Hospital, Munich, Germany

**Keywords:** standardized patient, medical interviewing, virtual consultation, digital teaching, WebEncounter

## Abstract

**Background: **Due to the pandemic-related restrictions in classroom teaching at the medical faculties of the LMU Munich and the University of Basel, teaching methods with standardized patients (SPs), were shifted to a digital, web-based format at short notice as of April 2020. We report on our experiences with the WebEncounter program, which was used for the first time in German-speaking countries. The program enables one-to-one encounters between SPs and students. Students receive an invitational email with brief instructions and background information on the case. SPs use case-specific criteria that are compliant with the learning objectives for digital evaluation during the encounter. A feedback session takes place immediately following the encounter. The SPs address the didactically relevant sections and can illustrate them with the corresponding video sequences. Finally, the students receive the links to the video recordings of the encounter and the feedback unit by email.

**Project description: **The aim of this pilot study was to analyze the practicability of the program and its acceptance by students and SPs. In addition, we examined whether the operationalization of the learning objectives in the form of assessment items has an impact on the content and thematic development of courses in the area of doctor-patient communication.

**Methods: **To implement the program, patient cases previously tested in communication seminars in Munich and Basel were rewritten and case-specific evaluation criteria were developed. SPs were trained to use the program, to present their patient figure online and to give feedback. The experience of those involved (faculty, SPs and SP trainers, students) in implementing the program was documented at various levels. The frequency and causes of technical problems were described. Student results on the patient cases and on the feedback items were collected quantitatively and, where possible, supplemented by free-text statements.

**Results: **Data from 218/220 students in Basel and 120/127 students in Munich were collected and evaluated. Students were very satisfied with the patient cases, the encounter with the SPs and their feedback: 3.81±0.42. SPs experienced the training as an increase in their competence and the structured feedback as particularly positive. The training effort per SP was between 2.5 and 4 hours. The results show predominantly normally-distributed, case-specific sum scores of the evaluation criteria. The analysis of the individual assessment items refers to learning objectives that students find difficult to achieve (e.g. explicitly structuring the conversation).

Problems in the technical implementation (<10 percent of the encounters) were due mainly to the use of insufficient hardware or internet connection problems. The need to define case-specific evaluation criteria triggered a discussion in the group of study directors about learning objectives and their operationalization.

**Summary: **Web-based encounters can be built into the ongoing communication curriculum with reasonable effort. Training the SPs and heeding the technical requirements are of central importance. Practicing the virtual consultation was evaluated very positively by the students – in particular, the immediate feedback in the protected dialogue was appreciated by all involved.

## Zusammenfassung

**Hintergrund: **Aufgrund der Pandemie-bedingten Einschränkungen im Präsenzunterricht wurden an den Medizinischen Fakultäten der LMU München und an der Universität Basel kurzfristig ab April 2020 Lehrformen mit Schauspielpatienten (SPs) auf ein digitales, webbasiertes Format umgestellt. Wir berichten über unsere Erfahrungen mit dem erstmals im deutschsprachigen Raum eingesetzten Programm WebEncounter. Das Programm ermöglicht 1:1 Kontakte zwischen SPs und Studierenden, die mit der Einladungsmail Kurz-Instruktionen zum Fall und Hintergrundinformationen erhalten. SPs benutzen Lernziel-konforme und Fall-spezifische Kriterien, die sie während des Gespräches digital bewerten. Unmittelbar nach dem Gespräch findet eine Feedback-Runde statt, in der SPs didaktisch relevante Abschnitte aufgreifen und mit den entsprechenden Video-Sequenzen unterlegen können. Abschliessend erhalten Studierende die Links zu den Video-Aufzeichnungen des Gesprächs und der Feedback-Einheit per Mail.

**Projektbeschreibung: **Ziel dieses Pilotversuchs war es, die Praktikabilität des Programms und seiner Akzeptanz bei Studierenden und SPs zu analysieren. Darüber hinaus sollte untersucht werden, ob die in dem Programm vorgeschriebene Operationalisierung der Lernziele in Form von Beurteilungs-Items Auswirkungen auf die inhaltliche und thematische Entwicklung von Lehrangeboten im Bereich der Arzt-Patient-Kommunikation hat.

**Methodik: **Zur Implementierung des Programms wurden in München und Basel bereits in Kommunikationsseminaren erprobte Patientenfälle umgeschrieben und Fall-spezifische Bewertungskriterien entwickelt. SPs wurden auf den Umgang mit dem Programm, die Online-Darstellung ihrer Patientenfigur und das Feedback-Geben geschult. Erfahrungen bei der Implementierung des Programms wurden auf den verschiedenen Ebenen der Beteiligten (Fakultät, SPs und SP-Trainer, Studierende) erfasst. Häufigkeit und Ursachen technischer Probleme wurden beschrieben. Ergebnisse der Studierenden auf Ebene der Patientenfälle und auf Ebene der Feedback-Items wurden quantitativ erfasst und wo möglich durch Frei-Text-Aussagen ergänzt.

**Ergebnisse: **Daten von 218/220 Studierenden in Basel und 120/127 Studierenden in München wurden erhoben und ausgewertet. Studierende waren mit den Patienten-Fällen, dem Kontakt mit den SPs und mit ihrem Feedback sehr zufrieden: 3,81±0,42. SPs haben die Schulungen als Zuwachs in ihrer Kompetenz und das strukturierte Feedback als besonders positiv erlebt. Der Schulungsaufwand pro SP betrug zwischen 2,5 und 4 Stunden. Die Ergebnisse zeigen überwiegend normal-verteilte, Fall-spezifische Summen-Scores in den Bewertungskriterien. Die Analyse der einzelnen Bewertungs-Items verweist auf Lernziele, deren Erreichen Studierenden schwerfällt (z.B. explizites Strukturieren des Gespräches).

Probleme in der technischen Umsetzung (<10 Prozent der Gespräche) waren überwiegend auf den Einsatz von ungenügender Hardware oder auf Verbindungsprobleme im Internet zurückzuführen. Die Notwendigkeit, Fall-spezifische Bewertungskriterien zu definieren, hat in der Gruppe der Kurs-Verantwortlichen eine Diskussion über Lernziele und ihre Operationalisierung angestossen.

**Zusammenfassung: **Webbasierte Gespräche lassen sich mit vertretbarem Aufwand in ein laufendes Kommunikationscurriculum einbauen. Schulungen der SPs und die Beachtung ausreichender technischer Voraussetzungen sind von zentraler Bedeutung. Das Üben der virtuellen Sprechstunde wurde von den Studenten sehr positiv gewertet – insbesondere das unmittelbare Feedback im geschützten Zwiegespräch wurde von allen Beteiligten geschätzt.

## Introduction

In the course of the “Corona crisis”, face-to-face encounters with students in university classroom settings, particularly with standardized patients (SPs), were prohibited. We take this opportunity to report on our experiences with a program, not previously used in the German-speaking countries, which despite such bans enables the use of SPs via a web-based platform: WebEncounter [https://enhancedlearn.azurewebsites.net/]. WebEncounter was developed at Drexel Medical School in Philadelphia, USA, and enables face-to-face encounters between students and SPs via the internet. Several publications from the English-speaking countries demonstrate the benefits of this and similar learning and teaching aids in the training of students and in the further training of residents and nurses [1], [2], [3], [4], [5], [6]. The program belongs to the group of teaching aids, which are usually used as part of blended learning, combining direct interactions among students in small groups or with real patients and internet-based computer-aided learning.

The use of SPs and their benefits in medical teaching is well-documented [7], [8], [9], [10] and common at most German-speaking universities [11].

One particular feature of using SPs, especially in the area of training communication and social skills, is the possibility of immediate "patient" feedback after a learning unit [12], [13]. The effectiveness of feedback depends on how closely it relates to the behavior the student just demonstrated and whether it refers to a standard of desirable behavior that is familiar to the student. The first point relates to the implementation of the feedback and the second to its content. With the aim of a close temporal succession of behavior and feedback, a procedure was developed under the term “Rapid Cycle Deliberate Practice” that uses the advantages of structured, timely and concrete feedback on the learning success (e.g. [14], [15]). In order for the feedback recipient to know what he or she should do differently next time, it must be clear what the desired behavior is; the student – and the teacher – must be familiar with such a standard. Monica de Ridder et al. [16], emphasize this when they write: “Feedback is specific information about the comparison between a trainees’ observed performance and a standard, given with the intent to improve the trainees’ performance.” Determining desirable behaviors as specifically as possible is not trivial [17]. First, the faculty is required to define and teach learning objectives in the communication curriculum so clearly that students (can) know what is expected of them. These learning objectives must then be applied to the specific case, i.e. formulated in such a way that they are represented in the conversation with the SP and that they can be assessed, if they occur. The persons who are to give the feedback must be trained according to these guidelines.

When implementing these requirements, particularly in the face-to-face encounter between students and SPs, a further factor plays a decisive role. If the feedback does not come from a third party (e.g. an expert or other student present), but is provided by the SP, then it is the SP’s responsibility to not only act like a credible patient, but also to simultaneously detect whether and when the given learning goals are achieved in the conversation. This double burden often means that the feedback does not refer to the concrete behavior of the student in a certain phase of the conversation, but more likely summarizes an overall impression.

The platform we are using for the first time in the German-speaking countries addresses these difficulties and counteracts them:

- The buttons for assigning the evaluation criteria on the screen are arranged in a way that they are easy for the SP to use during the encounter without losing contact with their role.
- It records the conversation and the time stamps that refer to moments in which learning objectives were more or less well achieved.
- It includes a feedback unit immediately following the encounter, in which SPs can import the video segments that they have marked with time stamps as didactically valuable (“teachable moments” [18], [19]).

## Project description

The aim of this pilot project is to evaluate the practicability of the new online platform and to obtain an impression of its acceptance by students and SPs. In addition, it seems interesting to get to know the particular challenges involved in implementing such a program and to find out whether the results can be fed back into the learning and teaching process by way of a feedback loop.

A classic implementation study in the strict sense (e.g. [20]) could not be carried out due to the urgency of finding ad hoc alternatives to classroom teaching. At the two institutions involved, the following tasks had to be completed within 4 or 6 weeks: training of persons as administrators (for data management; appointments for SP training and student and SP encounters), training of SPs, formulation of instructions for all involved; revision of the role scripts for patient cases and redefinition of the assessment criteria to facilitate structured feedback

Participation in the online encounter with SPs replaced the obligatory encounters with SPs in traditional classes planned in the curriculum. One online consultation hour in WebEncounter was agreed upon for each student.

The analysis and the evaluation of the pilot test was based on the project directors’ observations, the anonymized technical and content-related feedback from students and SPs retrieved from WebEncounter, as well as the students' performance.

## Methods

### Study participants

The investigation was conducted at both locations between April and June 2020. Data from students in the 3^rd^ year of study at the LMU in Munich (N=122; 73 f, 59 m) and from the 2nd year of study in Basel (N=220; 154 f, 66 m) were considered.

Students at both locations were randomly assigned to the individual case situations.

#### Data collection, data anonymization, consent to study participation 

Medical students in Basel are informed at the beginning of their studies that video recordings are a part of training and need be treated just as confidentially as patient information. In Bavaria, article 10, paragraph 3, clause 2, clause 1 BayHSchG allows the collection of evaluation data for the purpose of quality control in teaching. In addition, at the beginning of the summer semester while booking their courses for the summer semester 2020, the students were informed about the collection and use of data and video recordings. Students signed and gave their consent before booking the class.

At the University of Basel, research and publications that serve to improve teaching are permitted. In this particular case, the local ethics committee (EKNZ) decided that the study was not subject to the human research act art 2 and therefore no formal approval/assessment was necessary.The video files are saved under a randomly generated name (example: nejzh3aGquqK_1br4yt7qyr.mp4), to ensure that the real name of the video file including the user information cannot be read out.

The feedback from the students evaluated as part of the evaluation and the scoring-related evaluations are anonymized by WebEncounter for the respective query period or for each “patient” case as descriptive statistical statements summarized without personal data.

#### Development of the case descriptions

At both universities, the teams of actors and trainers selected those patient vignettes deemed most suitable for an online consultation from existing patient vignettes. The criteria were predominantly verbal references to the underlying diagnoses or processing styles of the patients, clear assignment of the case histories to specific learning goals and playability within 8 to 15 minutes.

In Basel, the following two cases were selected: suspicion of flour dust allergy and suspicion of sexually transmitted disease, including exclusion of HIV. Three cases were used in Munich: critical adherence to therapy for Hashimoto's thyroiditis, notification of an HIV infection and diagnosis of colon carcinoma.

A short case description (door instruction) was developed for each case. In traditional classes with SPs this is usually distributed in advance as brief information or attached to the door to the consultation room and informs the students about who they are going to meet and what their tasks are. In addition, the students received technical instructions as well as medical background information on the case scenario in advance, in order to prepare themselves.

#### Standardized patients

Twelve SPs took part in the course at each location (University of Basel: 8f /4m; age range 21 to 51 years; LMU Munich: 10f /2m; age range 25 to 65 years). 9/12 SPs in Basel and 6/12 SPs in Munich had previously worked with the cases used in WebEncounter in face-to-face lessons, the other SPs were trained in the content of the cases.

**Figure 1:**
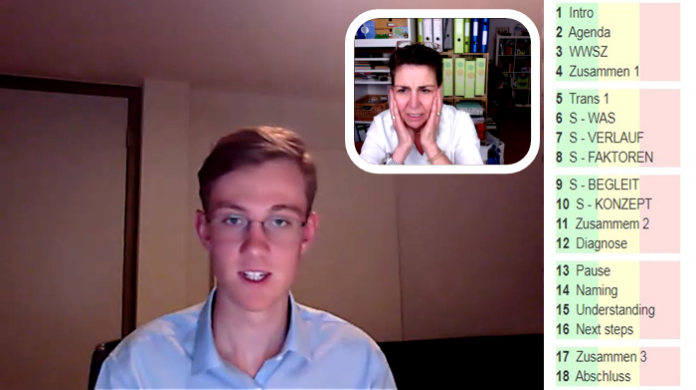
SP work screen during an online consultation in WebEncounter

In the training of the SPs, special features of case presentation via camera and with a limited field of view were discussed. Due to the limited field of view in the representation of the patient, non-verbal messages to the student which are expressed, for example, in changes in tension of the entire body had to be transferred to other non-verbal channels (e.g. tension of the upper body, covering face with both hands, etc.), facial expressions or verbal utterances (see figure 1 (Fig. 1)). During the conversation, the SP sees the student and herself in the upper right corner, as well as the assessment criteria.

The use of the assessment items was especially practiced in order to be able to give structured feedback. SPs already trained by the study leaders or the SP trainers took on the role of more or less talented students and then discussed their assessments with the SPs. If they had the impression that the feedback correctly reflected the different degrees of achievement of the learning objectives and that the SPs were also able to express themselves constructively to the “students”, the SPs were licensed for their respective case.

During their initial training and while working with the students, the SPs were continuously supported in the event of problems in handling the program. A debriefing was offered after a day of teaching. The average training effort per SP was 2.5 hours in Basel and 4 hours in Munich, and was conducted in an one-on-one setting and in small groups via ZOOM or directly via WebEncounter. During the teaching units, a backup was guaranteed by the SP trainers, the teaching staff (WL in Basel, AB and BO in Munich) and the program developer in Brazil.

In addition to the case-specific information, instructions for login into WebEncounter were sent out for each location. The SPs also received written explanations for evaluating the listed scoring items with text examples.

The module secretariat was responsible for coordinating the appointments. At the University of Basel, the two interlocutors were invited directly via the WebEncounter platform and at the LMU Munich – due to the initially high frequency of exchange – the invitations to the actors and students were sent by the module secretariat.

#### Implementation

The students received an invitation email from the program 2 to 5 days before their appointment with a link to the platform and background information as well as brief information on their case. The day before their appointment, they were reminded of the appointment either via WebEncounter or by the administrators

At the agreed time, students could use the link to join “their” SP who greeted them, explained the process, checked the technology, made sure that the students had read the case information and then had the consultation. Afterwards, feedback was offered in which the SPs gave specific feedback based on the assessment criteria, which they could back up with the corresponding video sequences. This option was rarely used by the students and the SPs. The discussions and the feedback units each lasted 12-15 minutes in Basel and 8 minutes in Munich. At the end, the students rated the case and the SP, and received a link to the recording of their consultation and their own feedback session. They were asked to make a note of any technical problems encountered during the session.

#### Description of the results

We report on the type and frequency of technical problems, the distribution of the total scores by the students per case, the distribution of the values in the individual scoring items and the mean values of the assessment of cases and SPs by the students. If possible, the sum scores are supplemented with examples from the students' and SPs’ comments.

## Results

### Observations from the implementation 

Results were obtained from 218 out of 220 participating students in Basel. In the two missing cases, it was not possible to record a meaningful video. In about 5% of the consultations (19/338) students experienced technical problems. The SPs reported a total of 9 events with technical problems (approx. 2.5%). In these cases, either a new appointment was made or the recording was repeated immediately, e.g. after students had switched from a poor WiFi connection to a local hotspot via their cell phone. When checking the entries and the login data, the impression was confirmed that problems were mainly due to poor connection quality. This was partly due to the fact that some students and SPs had ignored the instruction to connect to the program via a wired network access (LAN), or had dialed in with insufficient peripheral devices. In Basel, 12 SPs took part with between 15 and 23 consultations with students each and representing one case only.

Results are available from 120 of the 122 participating students in Munich. In the 2 missing cases, the recording of the consultation failed. From the student perspective, there were technical problems in 8 consultations, the SPs reported a total of 4 incidents with technical problems. In these cases, either a new appointment was made or the recording was repeated immediately. When checking the entries and the connection data, it was found that the problems in Munich were due mainly to the connection quality and the failure to follow the instructions. As in Basel, connection and operation problems could be explained by the fact that instructions for using a LAN connection were not followed or that unsuitable peripheral devices were used. In Munich, 12 SPs took part in classes with WebEncounter. The individual SPs completed between 3 and 20 WebEncounter consultations (median 7) with students, with between 3 and a maximum of 6 WebEncounter consultations being carried out in the individual appointments. The vast majority (9/12) of the SPs only represented one case; three SPs represented two cases.

The SPs’ feedback at the beginning of the training made it clear that they had to get used to the new “expert role”, i.e. to using the evaluation criteria; questions were clarified in the evening feedback sessions with the program managers and SP trainers. In the end, the feedback from the SPs on the use of the feedback criteria and on their own role and function was markedly positive. They particularly praised the intimacy of the one-on-one encounter, which made it easier for them to give personal feedback. In Basel in particular, it was emphasized that the students were much more open than in previous years, they actively requested detailed feedback and thanked them for it explicitly. The change in the role at the beginning of the encounter, from the “organizer”, who asks the student whether they had read the information, to ‘the patient’, was never considered to be a problem.

#### Sum scores of the individual cases

**Figure 2:**
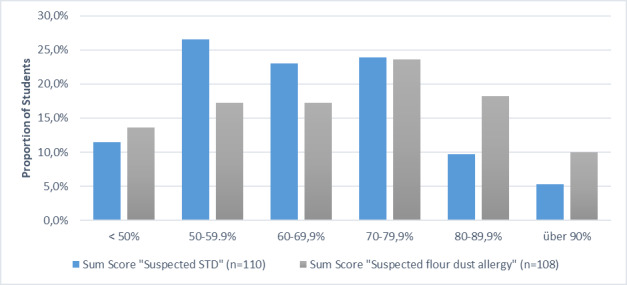
Distribution of students’ overall results by case at University of Basel

**Figure 3:**
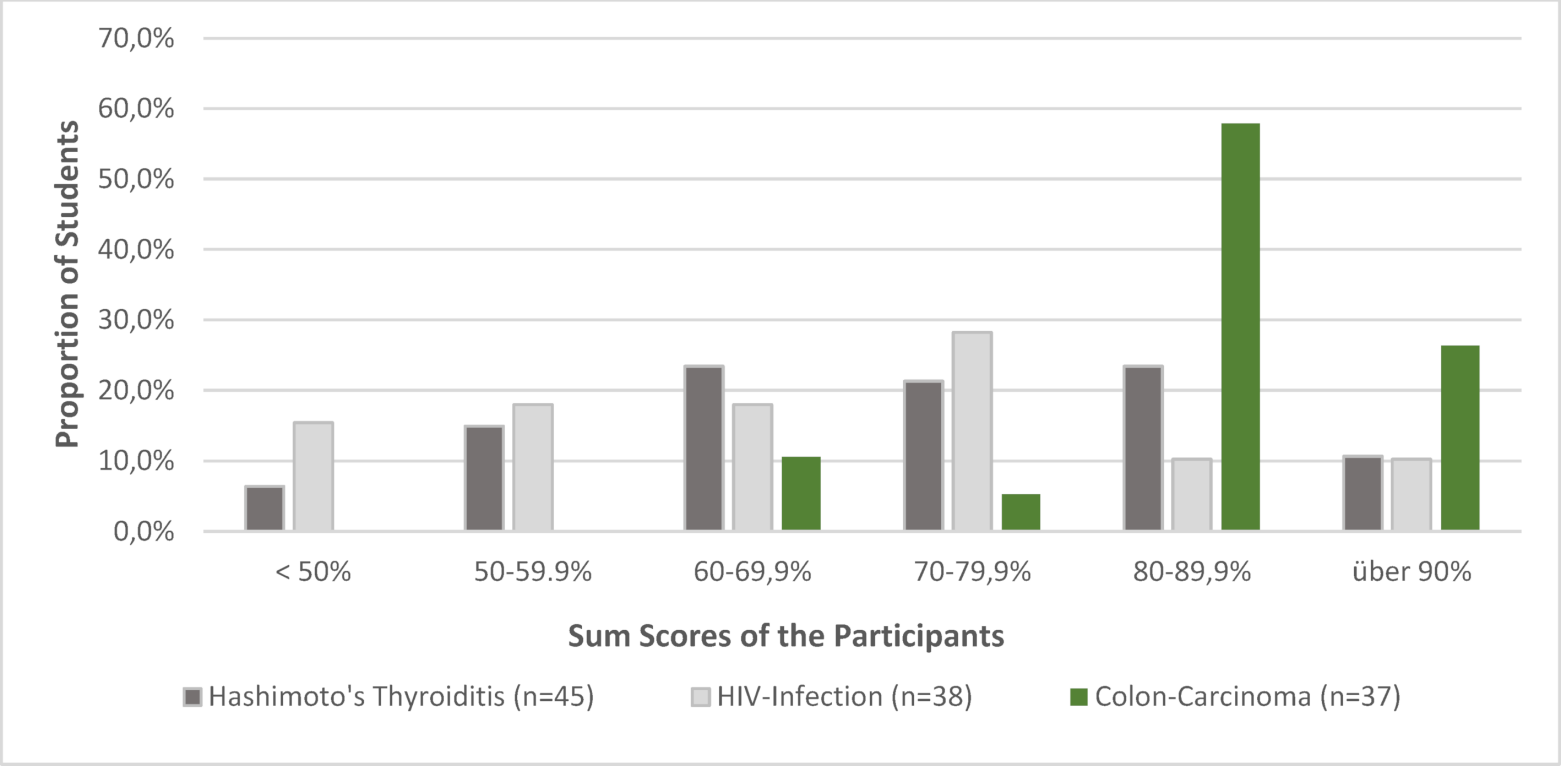
Distribution of sum scores at the LMU Munich

The individual evaluation items are assigned a score of 1 (not fulfilled), 3 (partially fulfilled) and 5 (fully fulfilled). The sum of all items results in the sum score (overall evaluation of the consultation as a percentage of points achieved) the distribution of which is shown in figure 2 (Fig. 2) and figure 3 (Fig. 3), separated by cases.

In most cases, there is an almost normal distribution of the range of variation with some significantly poorer students in the case of communicating a sexually transmitted disease (STD). The somewhat right-skewed distribution in the Munich scores of the case on communicating bad news (colon carcinoma) is also striking. As this study is a first attempt to use this program in German-speaking countries, all videos of the encounters in which students achieved below 30 percent of the possible score were viewed in order to rule out technical problems or unhelpful behavior on the part of the SPs. Neither could be verified. The responses from the SPs were correct and, in the perception of those responsible, corresponded to the behavior of the students. In the spirit of “closing the loop”, students in Basel – as has been the case in the last six years – whose score was two SDs below the class average, were invited to a refresher course in which the obviously problematic items were discussed in depth again and illustrated with examples. In Munich, students have the opportunity to implement the findings of the feedback in a further encounter with simulated patients in the following semester.

#### Sum scores of the individual items

**Figure 4:**
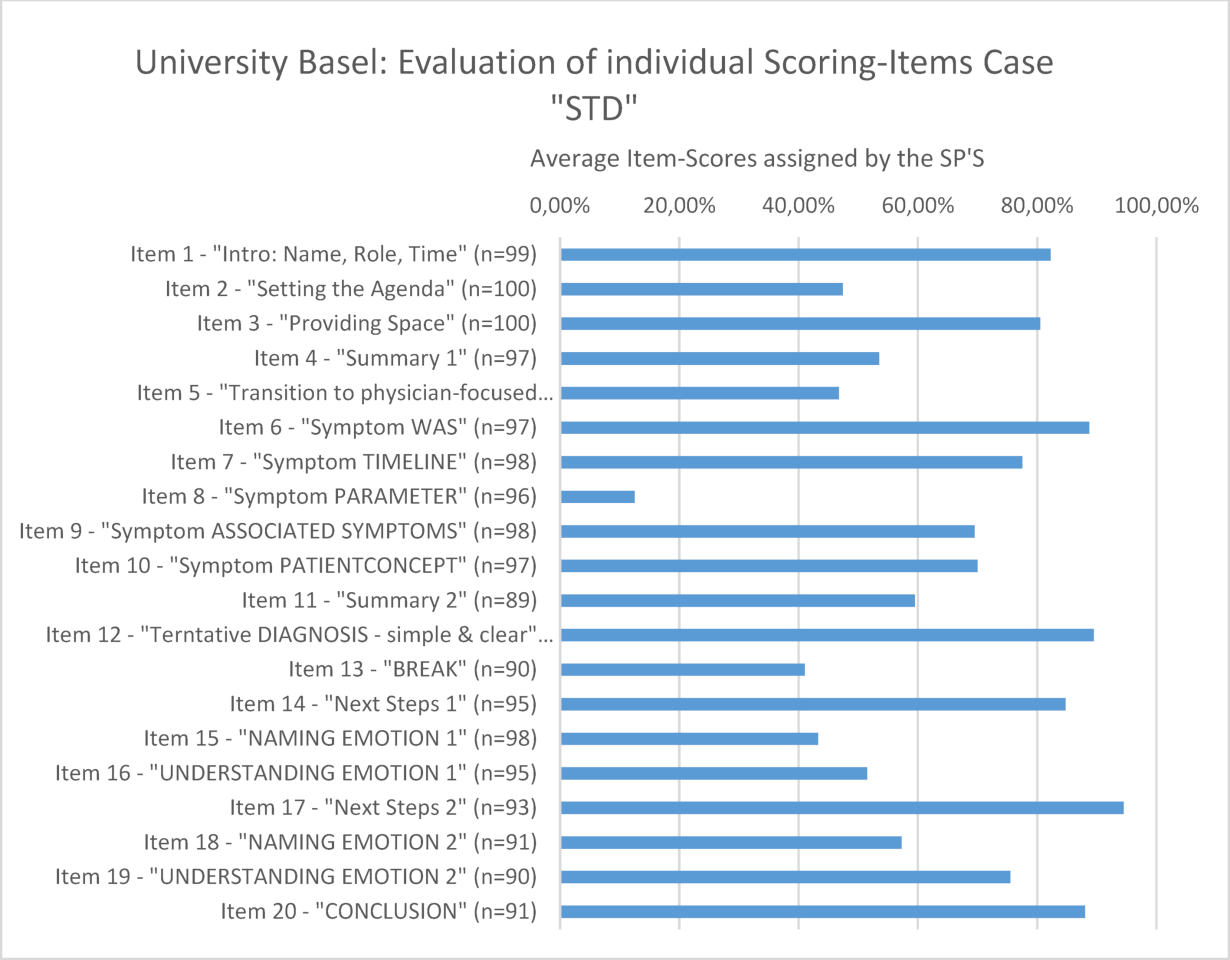
Example for the evaluation of individual scoring items

With regard to the individual items, it became clear that the students had difficulties with very specific items. This concerns, on the one hand, the items in which the explicit addressing of the conversation structure is depicted (clarifying the agenda for the conversation, explicit change from the patient-centered to the doctor-centered communication phase, announced summaries) and on the other hand, items concerning the systematic narrowing down of symptoms (see figure 4 (Fig. 4)). 

#### Student feedback on the SPs and the cases

**Figure 5:**
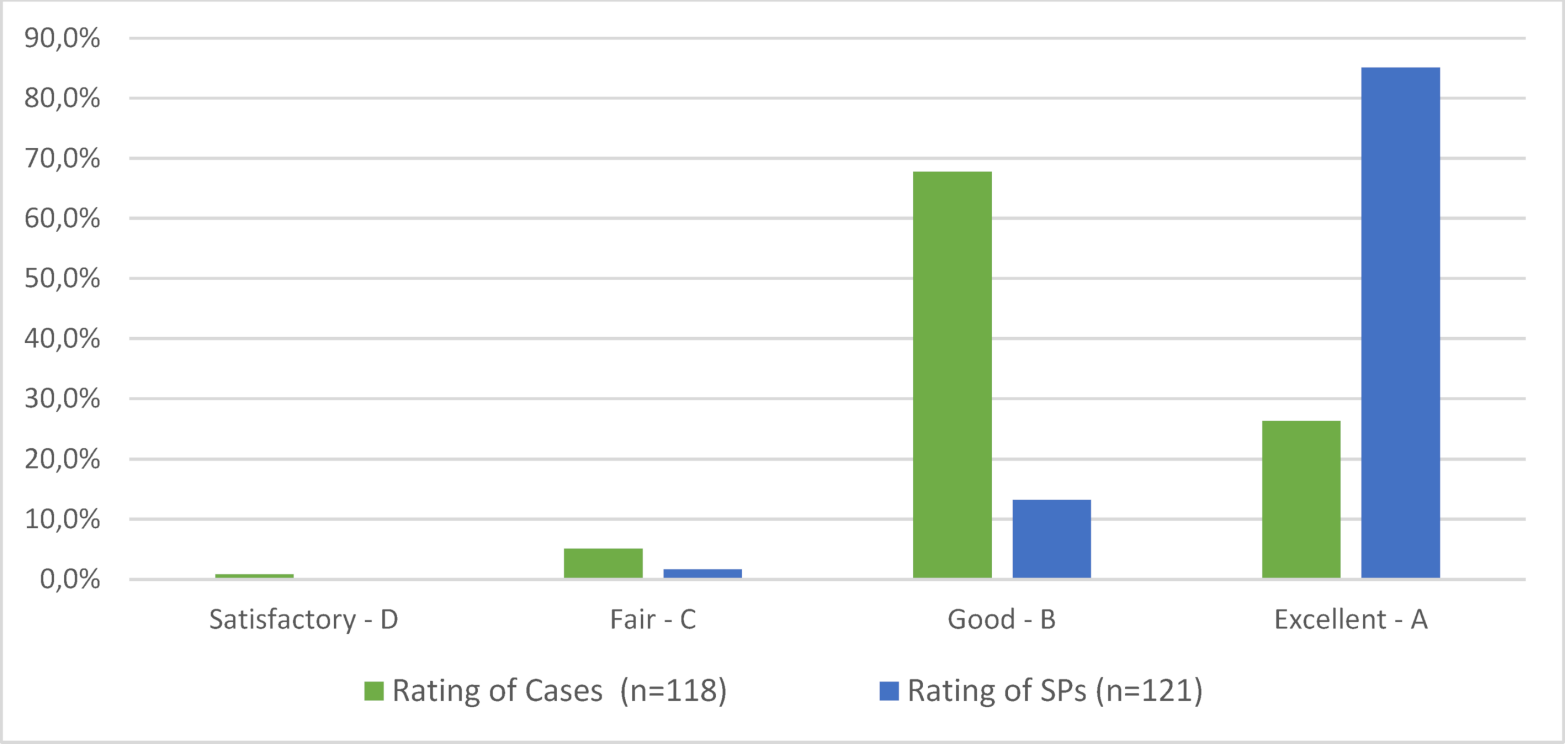
Evaluation of the cases and the SPs by the students

As figure 5 (Fig. 5) shows, both the cases and the SPs were rated very positively by the students. There was no negative feedback. However, of 120 students in Munich and 218 students in Basel, only around 35% completed the voluntary questionnaire at the end of the WebEncounter encounter. 

**Figure 6:**
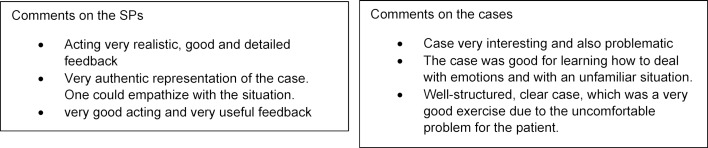
Selection of student comments

The qualitative feedback from the students confirms the positive overall impression. Students especially emphasized the constructive feedback (see figure 6 (Fig. 6)).

## Discussion

With regard to the technical implementation, the results available so far are predominantly positive. Especially “in times of Corona”, in which the internet has often been at the limit of its capacity, <10 percent technical problems, i.e. mostly connection-related, are a good result. To interpret the left-skewed results, in the case of suspicion of sexually transmitted diseases, we can draw on the feedback that the students gave in the voluntary zoom-meeting refresher course (100 of 220 students took part). They found it very difficult to have to talk about sexuality with an older man. For us, this aspect was important in terms of didactics and content. It gave rise to a discussion with the students about the fact that the special situation of a doctor allows or even requires different “access rights” to a person’s life than the situation in a private contact. With regard to the right-skewed distribution of the sum scores in the “colon carcinoma case”, we assume that the e-learning consultation, which preceded the online consultation, in which aspects and concepts of delivering bad news were discussed and refreshed, could have had an impact on the results.

The feedback from students on this form of learning is very encouraging. The SPs report that they also had the impression that students had benefited from the intimacy in the one-on-one setting. In doing so, they refer to their experiences in recent years, in which discussions with SPs occurred in a small group setting of five students in Basel. One of the students held the conversation and the others were supposed to try to identify “teachable moments”. SPs had criticized this teaching format because students in the second year course in Basel were often not ready or able to give each other concrete and constructive feedback, let alone discuss the SP’s feedback. In the discussion rounds with experts, in three to four groups of five after the SP encounters, the main criticism students expressed was that the exposure in the group during role-play with SPs and the feedback from the experts in the presence of the others was uncomfortable and embarrassing. Since the online consultation with WebEncounter was not tested against a face-to-face event at which students could speak to an SP on their own, it remains unclear whether the positive feedback regarding the intimacy of the situation was due to the relocation to the internet or the change from (small) group lessons to the one-on-one setting. In principle, the different teaching formats should not be played off against one another, but rather used according to their particular strengths and weaknesses and to meet the needs of the students in achieving different learning goals [21].

It was striking for us, those responsible for teaching, to what extent the special features of WebEncounter revealed inconsistencies with regard to the specific learning objectives in the area of medical interviewing within the faculty. This is because feedback items can only be formulated reliably and in a manner that is manageable for SPs if all participants agree that, for example, “explicit structure” is an essential element of a conversation and how explicit structure is addressed or implemented in a patient-centered manner in conversation. When the group responsible for the content within a faculty has agreed on the specific learning objectives, the evaluations of the SPs clearly indicate which learning objectives require further training. This localization of critical items enables “closing the loop” [22], [23], if – as is usual in Basel – a refresher course is offered, in which the critical items are discussed in depth. This year, 100 of the 220 students in Basel took part in this voluntary course offer as a virtual lecture.

Within our group and in discussions with SP trainers and SPs, there was critical objection in advance that this program could undermine the classic SP presence programs. In our opinion, however, this fear is not substantiated. The introduction of web-based teaching units does not mean less SP participation, but an upgrading of the work of SPs, whose area of competence is expanded to include the ability to give concrete and constructive feedback – and this without experts in the background.

An obvious point of criticism concerns the possible loss of depth of a real encounter if it is relocated into virtual space. We need to consider that SP-based teaching units take place in the presence of other students and an expert at most universities. The potential of atmospheric condensation by the intimacy of the direct encounter could be endangered by the publicness of this contact in small group lessons. Even if certain elements of the one-on-one encounter in reality are undoubtedly missing in web-based contacts, it remains to be seen whether this shortcoming is not offset by the clearly dyadic nature of the encounter.

Whether the definition of assessment criteria does not lead to the loss of the range of possible feedbacks is a further critical question. Under the best possible conditions, this will certainly be the case if, for example, the SPs are excellently trained in identifying behaviors that correspond particularly to the learning goals or in recognizing the personal strengths and weaknesses of the students in establishing and maintaining an empathic relationship. At the request of the SPs in Munich, we therefore included an open feedback criterion (“key moment”) that they could use, if necessary, if they noticed particularly conspicuous behavior. However, this feedback item was rarely used, which indicates that the existing criteria were sufficient. A fundamental caveat concerns the validity of feedback from the personal perception of experts or SPs: the literature shows that those not directly affected – including SPs or experts – do not perceive the peculiarities of the relationship between patients and professionals in the same way as those actually affected themselves [24]. This has just been substantiated again in a recent study on the perception of empathy [25], which showed that patient perceptions predict a reduction in fear and satisfaction through conversation with a high degree of accuracy, while expert judgments have no predictive quality and, moreover, are not related to patient perceptions.

In summary, previous experience with WebEncounter shows that this program is perceived by students and SPs as an enrichment to previous forms of teaching. In Basel, it will be used in the second year course in the next academic years. In Munich, WebEncounter is to be used as a supplement to face-to-face teaching in the 2020/2021 winter semester. Other possible uses are planned in courses on taking a medical history, when delivering bad news, when dealing with problematic explanatory concepts and in OSCE exams. 

In order to be able to further examine and analyze the benefits of this type of teaching and the possible uses of WebEncounter, comparisons of online one-on-one encounters and typical small group formats with the help of experts should follow. 

## Dedication

We dedicate this article to our program developer and colleague Christof Daetwyler, MD, who passed away unexpectedly last December. He was a wonderful example of student- and user-centered communication. His readiness to respond to our wishes and his patience in dealing with members of the working groups in Munich and Basel, who are not always tech-savvy, impressed us all.

## Acknowledgements

Special thanks are due to the actresses and actors who were our standardized patients and the trainers, who quickly adapted to the new situation with great enthusiasm. Furthermore, we wish to express our gratitude for Claudia Steiner and Kuno Steiner for the English translation and the list of references.

## Funding

- Basel: The project was supported with CHF 4,000 as part of the “promotion of innovative teaching”.
- Munich: The additionally required license costs and some technical devices were financed through the special Corona budget of the central university administration.

## Competing interests

The authors declare that they have no competing interests.
